# Integrated Analysis of Gene Expression, SNP, InDel, and CNV Identifies Candidate Avirulence Genes in Australian Isolates of the Wheat Leaf Rust Pathogen *Puccinia triticina*

**DOI:** 10.3390/genes11091107

**Published:** 2020-09-21

**Authors:** Long Song, Jing Qin Wu, Chong Mei Dong, Robert F. Park

**Affiliations:** Plant Breeding Institute, School of Life and Environmental Science, Faculty of Science, The University of Sydney, Sydney 2006, NSW, Australia; long.song@sydney.edu.au (L.S.); jingqin.wu@sydney.edu.au (J.Q.W.); chongmei.dong@sydney.edu.au (C.M.D.)

**Keywords:** *Puccinia triticina*, avirulence gene, next-generation sequencing, single nucleotide polymorphism, copy number variation

## Abstract

The leaf rust pathogen, *Puccinia triticina* (*Pt*), threatens global wheat production. The deployment of leaf rust (*Lr*) resistance (R) genes in wheat varieties is often followed by the development of matching virulence in *Pt* due to presumed changes in avirulence (Avr) genes in *Pt*. Identifying such Avr genes is a crucial step to understand the mechanisms of wheat-rust interactions. This study is the first to develop and apply an integrated framework of gene expression, single nucleotide polymorphism (SNP), insertion/deletion (InDel), and copy number variation (CNV) analysis in a rust fungus and identify candidate avirulence genes. Using a long-read based *de novo* genome assembly of an isolate of *Pt* (‘Pt104’) as the reference, whole-genome resequencing data of 12 *Pt* pathotypes derived from three lineages Pt104, Pt53, and Pt76 were analyzed. Candidate avirulence genes were identified by correlating virulence profiles with small variants (SNP and InDel) and CNV, and RNA-seq data of an additional three *Pt* isolates to validate expression of genes encoding secreted proteins (SPs). Out of the annotated 29,043 genes, 2392 genes were selected as SP genes with detectable expression levels. Small variant comparisons between the isolates identified 27–40 candidates and CNV analysis identified 14–31 candidates for each Avr gene, which when combined, yielded the final 40, 64, and 69 candidates for *AvrLr1*, *AvrLr15,* and *AvrLr24*, respectively. Taken together, our results will facilitate future work on experimental validation and cloning of Avr genes. In addition, the integrated framework of data analysis that we have developed and reported provides a more comprehensive approach for Avr gene mining than is currently available.

## 1. Introduction

Leaf rust, caused by *Puccinia triticina* (*Pt*), is the most widespread rust disease of wheat and the most damaging biotic stress of wheat globally, causing losses of around 3.25% globally [1]. Severe regional epidemics of the disease have occurred in many wheat growing areas including Western Australia in 1999 [2], and Kansas, USA, in 2007 where it caused 14% yield losses [3]. The deployment of leaf rust (*Lr*) resistance (R) genes in wheat varieties is the most effective and economical method for reducing yield losses and ensuring adequate quantities of pesticide-free food. However, *Pt* has great capacity to evolve virulence matching resistance in wheat cultivars to render them susceptible [4], as exemplified by the annual report of more than 50 virulence phenotypes detected annually in the United States [5]. In addressing this challenge, it is crucial to get a better understanding of the genetic basis of wheat-rust interactions at the molecular level.

Based on the gene-for-gene hypothesis [6], a model at the molecular resolution with two layers of plant immunity was proposed for plant-pathogen interactions [7]. In the first layer, pathogen-associated molecular patterns (PAMPs) are recognized by plant pattern recognition receptors (PRRs), resulting in PAMP-triggered immunity (PTI). In the second layer, a pathogen may breach PTI by deploying effectors (secreted proteins) that modify plant metabolism and defense response, and in turn, the plant host may evolve resistance proteins that specifically recognize pathogen effectors either directly [8] or indirectly [9] and result in effector-triggered immunity (ETI). Compared to PTI, ETI manifests as a hypersensitive response (HR) involving localized cell death and is more rapid and robust. However, the host ETI response can be evaded by a pathogen through modification of avirulence (Avr) genes, with the selection force driving the coevolution of R genes in plants and Avr genes in pathogens.

Identifying Avr genes in *Pt* and the corresponding R genes in wheat is crucially important in understanding the genetic basis of wheat–rust interactions and in developing new approaches and diagnostic tools to reduce the losses caused by *Pt*. Although more than 80 leaf rust R genes have been catalogued [10], no single Avr gene in *Pt* has been identified yet [11]. Despite the presence of virulence for the R gene *Lr1* in Australia, this gene was used effectively for many years by combining it with other R genes for multiple gene resistance [12]. For the R gene *Lr15*, the frequencies of virulence are low in Australia but relatively common in most geographical areas [13]. Like *Lr1*, the R gene *Lr24* remains important in Australia because it can be combined with other R genes to provide protection against all known pathotypes of *Pt* [2]. Due to the usefulness of these three R genes, we undertook a comparative genomics approach to identify candidate avirulence genes matching each in *Pt*. Australia is isolated from other major world wheat growing regions and wheat infecting rust fungi do not undergo sexual recombination there due to lack of alternate hosts [2,14]. Mutations within the wheat rust fungi are well documented in Australia and long-term rust surveys in this region suggest that new pathotypes mainly arise from single-step mutations. Clonal lineages have arisen within the rust pathogen populations via the sequential addition of single virulence [15]. The major *Pt* lineage in Australia between 1988 and 2010 was derived from the founding pathotype 104–2,3,(6),(7),11 (isolate S423; referred to as Pt104) (Park, unpublished), first detected in Australia in 1984 [16]. For this study, we selected six clonally derived isolates within this Pt104 lineage, two isolates from a second lineage known as Pt53 [17], and four isolates from a third lineage Pt76 (Park, unpublished) for genomic comparisons.

Although difficulties in maintaining pure isolates of obligate biotrophs like *Pt* have hindered genetic studies, next-generation sequencing (NGS) technology holds the promise of accelerating biological research and discovery in rust genomics. Compared to traditional polymerase chain reaction (PCR)-based approaches of genetic studies towards limited targeted regions, NGS enables genome-wide identification of candidate avirulence genes by comparing wild and mutated DNA sequences. Multiple reference genomes are now publicly available for each of the three wheat rust pathogens *Pt*, *Puccinia graminis* f. sp. *tritici* (*Pgt*), and *Puccinia striiformis* f. sp. *tritici* (*Pst*) [18]. With the availability of reference genomes, advances have been made for all three wheat rust fungi in genome-wide studies for effector mining, including studies on *Pst* [19,20,21], *Pgt* [22], and *Pt* [23], which have detected a panel of candidate avirulence genes for functional validation. One earlier study identified 15 candidates for 14 Avr genes using gene expression and RNA SNP analysis [24]. Moreover, two recent studies successfully identified and validated two Avr genes *AvrSr50* [25] and *AvrSr35* [26] in *Pgt*. In contrast to an array of studies on *Pgt* and *Pst*, the whole-genome sequencing studies on *Pt* are limited [23,27].

In addition to the analysis of gene expression, single nucleotide polymorphisms (SNPs), and insertions/deletions (InDels) [21,23,27,28], copy number variations (CNVs) have been highlighted as a new and significant source of genetic polymorphism that contributes to phenotypic diversity such as virulence in diverse fungal species [29]. In fact, the contribution of CNVs to population genetic and phenotypic diversity has been exemplified by a range of fungal studies, including studies on yeast *Saccharomyces cerevisiae* (Ascomycota, Saccharomycetes) [30,31], the wheat pathogen *Zymoseptoria tritici* (Ascomycota, Dothideomycetes) [32], and the human fungal pathogen *Cryptococcus deuterogattii* (Basidiomycota, Tremellomycetes) [33]. It has also been noted that the degree of CNV in fungal populations is not always correlated with the degree of SNP variation, which indicated that CNV analysis could reveal an important yet hidden layer of genetic information independent of SNPs/InDels. Despite the important role of CNVs as aforementioned [29], genome-wide studies of CNV analysis for wheat rust fungi are lacking.

The present study is the first to attempt an integrated analysis of gene expression, SNP, InDel, and CNV in association with virulence phenotype to identify candidate avirulence genes in *P. triticina*. Sequencing was performed on DNA samples of 12 *Pt* isolates from three clonal lineages and RNA samples of three *Pt* isolates representing field-collected pathotypes that dominated *Pt* populations in all mainland states in Australia [15,16]. Using a published long-read-based genome assembly of Pt104 [27], a haplotype-phased genome of the founding isolate of the lineage Pt104, we generated SNP, InDel, and CNV profiles for each isolate followed by comparative analysis to identify 40, 64, and 69 candidates for *AvrLr1*, *AvrLr15*, and *AvrLr24*, respectively. This study not only provides important new resources for future research of *Pt* in Australia and beyond but also demonstrates a practical framework of integrated analysis from multiple genomic aspects to explore candidate avirulence genes.

## 2. Materials and Methods

### 2.1. DNA Sequencing

All isolates used in this study were identified in annual nationwide race surveys of pathogenicity in *Pt* in Australia (Park 2008; Park, unpublished data) and were curated in the Plant Breeding Institute Rust Collection, The University of Sydney, Australia. Each isolate was established from a single pustule from a region of low-density infection and increased on wheat plants of the susceptible variety Morocco. For rust infection, wheat plants were grown at high density (~25 seeds per 12 cm pot with compost as growth media) to the one leaf stage (~7 days) in a greenhouse microclimate set at 18–25 °C temperature and with natural day light. The identity and purity of each isolate was verified by pathogenicity tests with a set of host differentials. Mature spores were collected, dried, and stored at −80 °C. From dried dormant spores, DNA was extracted as described elsewhere [34] and sent to Novogene (Hong Kong, China) for Illumina short-read sequencing. TruSeq library of DNA samples for the 12 *Pt* isolates were constructed with a 150 bp paired-end and sequenced on a HiSeq X instrument (Illumina, San Diego, CA, USA).

### 2.2. RNA Sequencing

The three *Pt* isolates (S96, S108, and S473) and wheat cultivar Chinese Spring (without the resistance genes *Lr1*, *Lr15* and *Lr24*) were used for RNA sequencing. For each isolate, infected leaves were harvested at 3, 5, and 7 days after inoculation and immediately stored in liquid nitrogen. Samples were ground to a fine powder in liquid nitrogen and total RNA from rust pathogen and wheat leaf was extracted with the ISOLATE II RNA Mini Kit (Bioline, London, UK). After DNase treatment (Promega, Madison, WI, USA), RNA was purified by on-column DNase treatment and the quality was checked using the Bioanalyzer 2100 (Agilent, Santa Clara, CA, USA). About 10 μg of total RNA was processed with the Illumina mRNA-Seq Sample Preparation kit for library preparation. Each library was sequenced using the Illumina HiSeq 2500 platform (125 bp paired-end reads) at Ramaciotti Centre for Genomics (Sydney, Australia).

### 2.3. RNA-Seq Analysis for the Selection of Expressed Effectors

The quality of the raw data of RNA sequencing was assessed using FastQC v0.11.8 with default options. The data were trimmed with Trimmoatic v0.38 with parameters “LEADING:3 TRAILING:3 SLIDINGWINDOW:4:25 MINLEN:36.” After trimming, the quality of data was assessed again before mapping. The paired-end reads data of each isolate was mapped to Pt104 individually using STAR 2.7.0 [35]. The alignments were selected using SAMtools 1.6 view command with parameters “-q 10.” The generated BAM files were sorted and indexed using SAMtools. From BAM files, the mapped reads were counted using the Bioconductor package Rsubread v2.0.1 [36], which generated the counts of mapped reads for each gene. Based on the gene annotation of the Pt104 assembly, effectors were predicted using EffectorP 2.0 [37], a machine learning classifier for fungal effector prediction. To validate the predicted effector gene, if the count values of mapped reads reached at least 10 in one or more of the three RNA-seq samples, this gene was regarded as being expressed and selected. Ortholog searches between Pt104 and *Pt* Race 1 genomes were performed using Proteinortho v5.16 (synteny mode) [38]. The localizations of effectors in plant cells were predicted using LOCALIZER v1.0.4 [39] and ApoplastP v1.0.1 [40].

### 2.4. Quality Assessing, Trimming, and Mapping for Whole-Genome Sequencing

The quality of original pair-end reads data were assessed using FastQC v0.11.8 [41] with default options. The data were trimmed with Trimmomatic v0.38 [42] with parameters “LEADING:3 TRAILING:3 SLIDINGWINDOW:4:25 MINLEN:36.” The quality of data was assessed again before mapping. The pair-end reads data of each isolate was mapped to Pt104 assembly individually using bwa v0.7.17 [43] with the BWA-MEM algorithm. High quality alignments were selected using SAMtools 1.6 [44] view command with parameters “-q 30.” The generated BAM files were sorted and indexed using SAMtools for subsequent data analysis. The mapping information summary was generated using SAMtools and BEDTools v2.25 [45].

### 2.5. SNP and InDel Calling and Comparative Genomic Analysis

From the indexed and sorted BAM files, small genomic variants were called using GATK v3.8.0 [46]. For each BAM file, regions around InDels were identified and realigned using GATK RealignerTargetCreator and IndelRealigner [47]. Genome-wide variant calling per isolate was performed using GATK HaplotypeCaller, and joint genotyping on all isolates was performed using GATK GenotypeGVCFs [48]. Variants were filtered using vcffilter command of vcflib [49] with parameters “DP > 10 and QUAL > 20.” From the virulence profiling of 12 *Pt* isolates against 21 R genes, a heatmap with a dendrogram was inferred and visualized using the Bioconductor package ComplexHeatmap v2.2.0 [50]. Based on the genomic variants detected, a phylogenetic tree of the 12 *Pt* isolates were inferred using SNPhylo ver. 20160204 [51] and visualized by the Bioconductor package ggtree v2.0.4 [52]. The R package circlize v0.4.8 [53] was used to create the Circos plot of genomic landscape for small genomic variants.

Using the Bioconductor packages VariantAnnotation v1.32.0 [54], rtracklayer v1.36.0 [55], and GenomicFeatures v1.38.2 [56], the identified genomic variants including SNPs and InDels were annotated and the functional impact of the variants were predicted and classified into major categories such as synonymous (SY), nonsynonymous (NSY), and frameshift across all isolates. For the identification of candidate avirulence genes, the differential variants with functional impact located within SP gene within each pairwise comparison (avirulence vs. virulence) were selected, which were examined across pairwise comparisons targeting at the same Avr gene, and those genes showing presence across all pairwise comparisons were considered as the final candidate avirulence genes.

### 2.6. Copy Number Variation Analysis

CNV analysis was performed using the Bioconductor package cn.MOPS 1.32.0 [57]. By using a mixture of Poisson models for read depths across multiple isolates in each genomic region, cn.MOPS can remove the effect of read depth variation along chromosomes and gain high sensitivity and a lower false positive rate. The BAM files were converted into read count matrices using the function getReadCountsFromBAM of cn.MOPS with the parameter “WL = 300.” The value of parameter WL (window length) was carefully chosen to make sure that on average, about 100 reads, were contained in each segment. The R package circlize v0.4.8 [53] was used to create the Circos plot of genomic landscape for CNVs. The example of a CNV was visualized using cn.MOPS. The differential CNVs were identified by comparing avirulent and virulent isolates, which were then inspected for the presence of the effector-encoding genes.

## 3. Results

### 3.1. Secretome Prediction by EffectorP and Validation by RNA Sequencing

The current study used our recently reported Pt104 genome assembly as the reference, which is long-read based and represents the best quality *Pt* genome assembly available to date [27]. Six presumed mutational derivatives of the isolate used to generate this reference genome assembly, two from a second lineage (the Pt53 lineage) and four from a third lineage (the Pt76 lineage), were selected for this study. The 12 isolates showed various virulence/avirulence profiles on catalogued resistance genes (Supplementary File S1).

EffectorP 2.0 [37] was used to predict secreted protein (SP) encoding genes from among the 29,043 genes previously annotated for the Pt104 genome [27]. This identified 5325 genes encoding potential effectors, each with an effector probability. To further validate the predicted SPs, RNA sequence data for two isolates that were avirulent on *Lr1*, *Lr15*, and *Lr24* (S96 and S108) and one isolate that was avirulent on *Lr15* and *Lr24* (S473) were used to select the SPs with detectable expression. After quality trimming, 77.2–129.1 million reads were obtained (Supplementary File S2). The trimmed RNA-seq data for each sample were mapped to the Pt104 genome individually. On average, 17.6–49.6 million reads (14.0–40.8% of total reads) could be successfully mapped to the reference; the remaining unmapped reads were dropped as they were largely from the wheat host transcriptome. For each SP gene, if the count values of the mapped reads were no less than 10 in at least one of the three samples used for RNA-seq, then this SP gene was regarded as being expressed and selected. Out of 5325 predicted SPs, 2392 were thus selected for subsequent effector mining (Supplementary File S3).

### 3.2. Mapping Whole-Genome Sequencing Data

**Figure 1:**
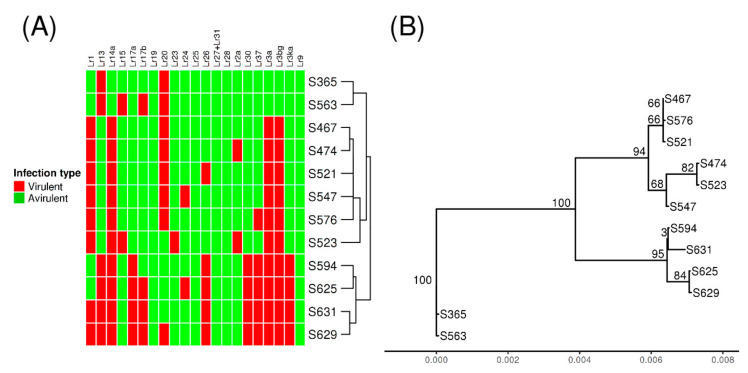
Heatmap and dendrogram based on pathogenicity (virulence/avirulence) of 12 *Puccinia triticina (Pt)* isolates, and a phylogenetic tree based on the identified single nucleotide polymorphism (SNPs) across the 12 *Pt* isolates. (**A**) The dendrogram separates the 12 isolates into three distinct clades. (**B**) The phylogenetic tree also separates the 12 isolates into three clades, consistent with those of the dendrogram. The numbers shown on the tree branches are the percentage of bootstrap replicates (1000) supporting the cluster.

To identify candidates for *AvrLr1*, *AvrLr15,* and *AvrLr24*, six *Pt* isolates from the Pt104 lineage (S467, S474, S521, S523, S547, and S576), two isolates from the Pt53 lineage (S365 and S563), and four isolates from the Pt76 lineage (S594, S625, S629, and S631) with contrasting virulence profiles were selected for whole-genome sequencing. The dendrogram depicting the virulence profiles of these isolates in Figure 1A illustrates the relatedness of these isolates based on virulence/avirulence profiles (Supplementary File S1), with three clades corresponding to the three clonal lineages.

After quality trimming, 66.3–96.1 million reads were obtained (Table 1). To genotype the isolates, each was individually mapped to the reference genome Pt104. Across the 12 isolates, between 56.8 and 87.9 million reads (75.0–93.1% of total reads) were mapped to the reference (Table 1). The percentage of mapped reads in the reference genome was 97.7–99.4%, which meant that almost all genes annotated in the reference could be genotyped for every isolate. The bam files generated from mapping were used for SNP, InDel, and CNV analysis as described in the following sections.

### 3.3. Genome-Wide Polymorphism and Phylogenetic Analysis

**Figure 2:**
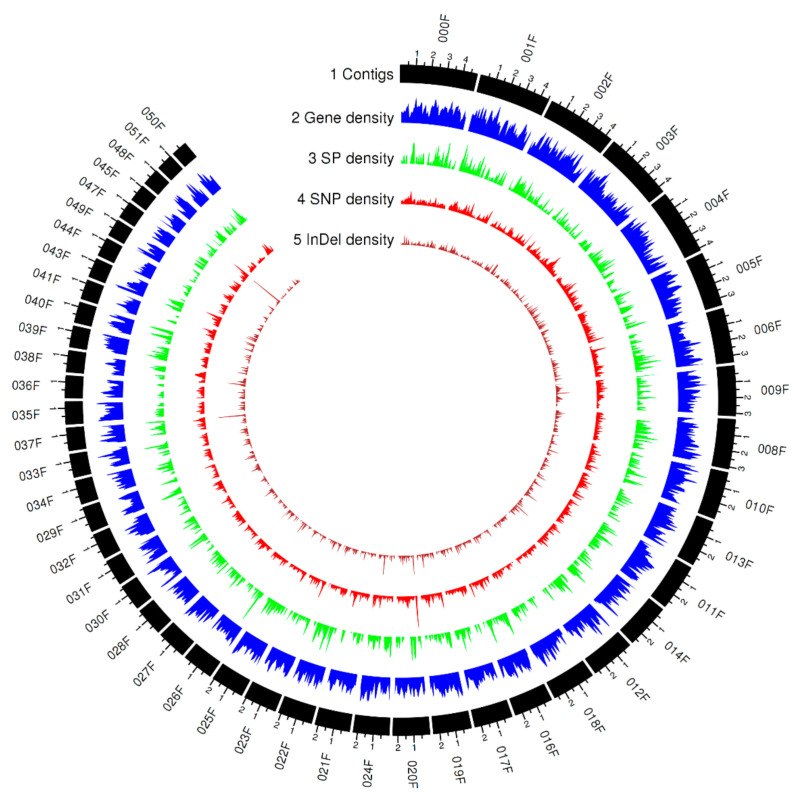
Genomic landscape of predicted genes, secreted proteins with detectable expression level, and genetic variations across 12 *Pt* isolates represented by the Circos plot of the top 48 contigs ranked by contig length (75.3% of the Pt104 genome). Tracks from outside to inside are: (1) contigs; (2)–(5) density of gene, secreted protein (SP), SNP (single-nucleotide polymorphism), and InDel (insertion or deletion) in nonoverlapping 100 kb windows. Each major tick on the contig track is for 1 Mb length.

A detailed view of genome-wide polymorphism was obtained by identifying small genomic variants including SNPs and InDels using GATK [48] and the bam files generated from the mapping as aforementioned. Between 525,308 and 686,935, variants were identified, with 72.4–93.2% of these being present in a heterozygous state (Table 2). The percentages of heterozygosity were similar within each clade but varied between clades, supporting the postulated relatedness of the isolates based on phenotypic studies. The genome-wide variant frequency was 2.9–3.9 variants/Kbp. The genomic landscape of predicted gene, secreted protein with detectable expression, and genetic variation across the 12 isolates represented by a Circos plot is shown in Figure 2. The numbers of SNP and InDel variants were 451,414–606,306 and 71,807–80,629, respectively, and the ratio of SNP/InDel was 6.1–7.6:1, indicating that most small genomic variants were SNPs.

Based on the SNPs detected across the whole-genome, a phylogenetic tree was inferred (Figure 1B). The topology of the phylogenetic tree was consistent with the putative relatedness of the isolates derived from the previous phenotype study (Figure 1A,B). Both the phylogenetic tree and the dendrogram derived from virulence profiles showed three distinct clades, and each clade comprised the same set of isolates.

### 3.4. Functional Impact of Small Genomic Variants

To evaluate the functional impact of small genetic variants, the variants were annotated using the Bioconductor package VariantAnnotation [54]. Of the total genomic variants identified, 79,896–104,269 (about 15.2%) were located within a coding region, covering 15,560–17,989 genes (Table 3). Variants within a coding region were classified into four types, namely, SY, NSY, frameshift (InDels causing frameshifts whose sizes were not divisible by three), and nonsense (premature stop codons). Among the 12 isolates, the average counts for each type as aforementioned were 27,806, 49,615, 7711, and 1379, respectively. Excluding SY variants, which did not result in an amino acid change, all other variants may have functional impact; all were, therefore, inspected further in the subsequent analysis of genomic pairwise comparisons. Interestingly, although the numbers of coding variants varied, the ratios of coding variants vs. total variants remained almost the same across the 12 isolates. This was also true for the ratios of nonsynonymous and synonymous variants vs. coding variants.

### 3.5. Small Genomic Variations Correlated with Avirulence/Virulence Phenotype

Paired comparisons between avirulent and virulent isolates were designed based on phylogenetic relatedness and contrasting pathogenicity for the resistance genes *Lr1*, *Lr15*, and *Lr24* (Figure 1). For *AvrLr1*, four paired avirulent versus virulent comparisons were selected: S594 vs. S629, S625 vs. S629, S594 vs. S631, and S625 vs. S631. Similarly, for *AvrLr24* and *AvrLr15*, four (S467 vs. S547, S474 vs. S547, S521 vs. S547, and S576 vs. S547) and three (S467 vs. S523, S474 vs. S523, and S365 vs. S563) paired comparisons were selected, respectively.

To detect candidates for each Avr gene, the SP genes covering differential variants with functional impact within each pairwise comparison were selected, then these selected SP genes were examined across pairwise comparisons and those showing presence across all pairwise comparisons were considered as candidate avirulence genes. For example, for *AvrLr15*, there were 451 differential variants located within 237 SP genes between S467 and S523, 414 differential variants within 239 SP genes between S474 and S523, and 214 differential variants within 89 SP genes between S365 and S563 (Supplementary File S4). Intersecting these three sets of genes resulted in 38 candidates for *AvrLr15*. Similarly, for *AvrLr1* and *AvrLr24*, genomic pairwise comparisons detected 27 and 40 candidates, respectively. For the candidates of *AvrLr1*, *AvrLr15*, and *AvrLr24*, 44–58% were identified by NSY, 5–22% by frameshift, and 33–38% by mixed types (e.g., a combination of NSY and frameshift) (Supplementary File S5).

### 3.6. Copy Number Variations across the Pt Isolates

**Figure 3:**
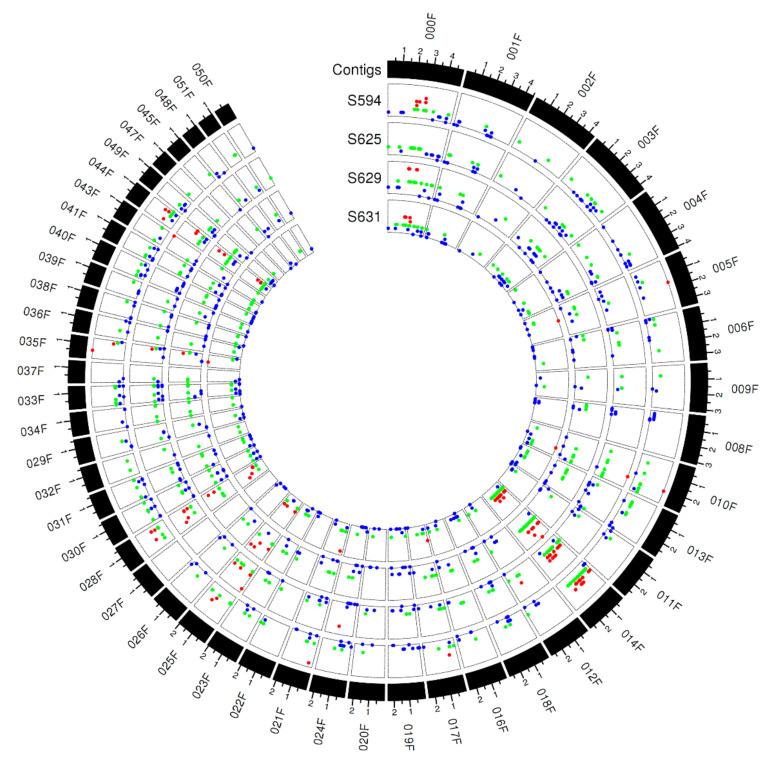
Genomic landscape of detected CNVs (copy number variations) for *Pt* isolates S594, S625, S629, and S631, which comprised the four isolates used to identify candidates for *AvrLr1*. Tracks from outside to inside are: (1) contigs and (2)–(5) CNV locations for S594, S625, S629, and S631. The height of each point represents the related copy number value. Red/green/blue, respectively, indicates the copy number is greater than, equal to, and less than 2. Each major tick on the contig track is for 1 Mb length.

Compared to small genomic variants (SNPs and InDels), genome-wide CNVs spanned larger regions and represent a different layer of genomic variation that may contribute critically to fungal pathogenicity. The Bioconductor package cn.MOPS, a central CNV identification tool capable of detecting the digitized copy number of genomic regions and simultaneous analysis of multiple isolates, was used to examine genome-wide duplications and deletions across the 12 *Pt* isolates [57]. A total of 307–2235 CNVs were detected across these isolates (Table 4). The CNVs detected in S594, S625, S629, and S631 are depicted in Figure 3. The total size of CNVs had a broad range from 1,997,415 to 10,609,561, corresponding to 1.4% and 7.5% of the reference assembly. The total numbers and sizes of CNVs were similar within each clade and varied between clades across the 12 isolates (Figure 1, Table 4), which again confirmed the putative relatedness of the isolates. Out of the total CNVs identified, 44.8–50.2% spanned gene-encoding regions and there were 385–2039 CNV-spanned genes (i.e., genes overlapped by CNVs) per genome. Out of the total CNV-spanned genes in each isolate, 16–69 were predicted SP-encoding genes.

### 3.7. Copy Number Variations Correlated with Avirulence/Virulence Phenotype

**Figure 4:**
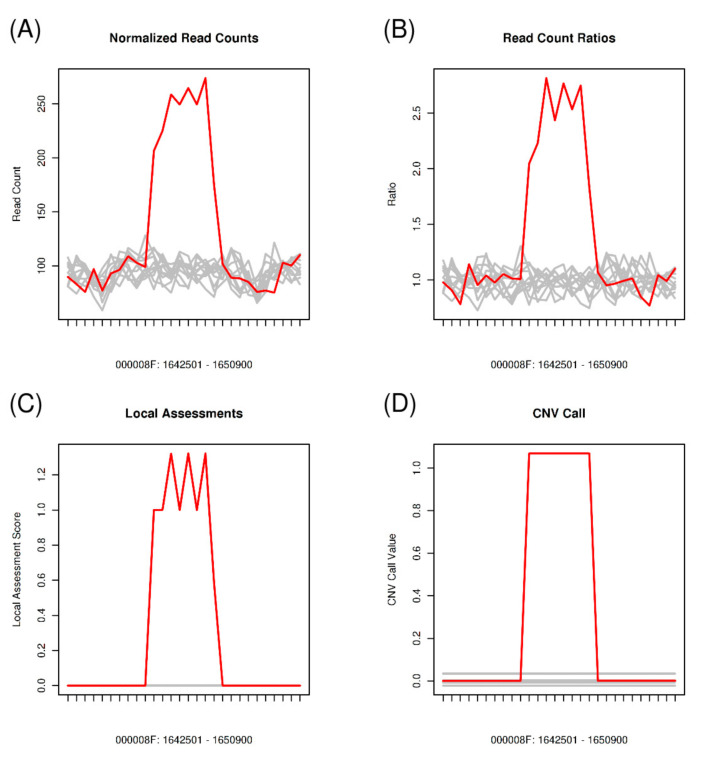
An illustration of a CNV detected at region 1,645,501–1,647,900 of contig 000008F. Isolate S547 (the red line) had the copy number 5 (copy number gain), and the other 11 isolates (grey lines) had the normal copy number 2. The candidate GN104ID162_006610 for *AvrLr24* was identified by this CNV (Supplementary File S6). The *x*-axis represents the genomic position, and the *y*-axis represents (**A**–**D**) normalized read counts, read count ratios, local assessment scores, and CNV calls produced by the segmentation algorithm [57].

To identify genes spanned by differential CNVs that may correlate with a given Avr gene, we compared the CNVs using the same isolate groups constructed for SNP and InDel comparisons as aforementioned. For *AvrLr1*, the CNV comparison was performed between isolates S594, S625, S629, and S631, revealing the presence of 511 differential CNVs in these four isolates. Out of these differential CNVs, 198 CNVs overlapped with gene coding regions, which affected 363 genes in total. Of these 363 CNV-affected genes, 14 genes were SPs, which were identified as the candidates for *AvrLr1* (Supplementary File S6). Similarly, for *AvrLr24*, 707 differential CNVs were found across isolates S467, S474, S521, S576, and S547, and 323 CNVs spanned the gene coding regions. A total of 31 SP genes harbored by these differential CNVs were identified as candidates for *AvrLr24*. A CNV that overlapped an SP gene is illustrated in Figure 4. For *AvrLr15*, differential CNVs were derived from two subgroups, S467, S474, and S523 as one subgroup and S365 and S563 as the other, and 1003 differential CNVs were identified. A total of 29 SP genes were detected as the candidates for *AvrLr15* (Supplementary File S6).

### 3.8. Final Candidate Avirulence Genes and Their Biological Annotations

By integrating the results derived from comparisons of small genomic variants and CNVs, we identified 40, 64, and 69 candidates for *AvrLr1*, *AvrLr15,* and *AvrLr24*, respectively (Table 5). A previous transcriptome study using *Pt* Race 1 as the reference genome reported one candidate (PTTG_25509) for *AvrLr24* [24]. However, no orthologs of the *Pt* Race 1 PTTG_25509 gene were found in Pt104. The likely reason for this is the differences between the short-read based genome assembly of *Pt* Race 1 for an American isolate and the long-read based genome assembly of Pt104 for an Australian isolate. Using LOCALIZER v1.0.4 [39] and ApoplastP v1.0.1 [40], the locations of 17–34 candidate effectors in the plant cell could be predicted as following: 4–13 in the apoplast, 2–7 in the chloroplast, 2–3 in the mitochondrion, 6–10 in the nucleus, and 2–6 in multiple locations (e.g., in chloroplasts, mitochondria, and nuclei) (Supplementary File S7). The biological functions of candidate avirulence genes were further inspected based on the Pt104 annotation using databases such as InterProScan (a database of protein families, domains and functional sites), MEROPS (peptidase database), and transcription factor (TF) families (Supplementary Files S8 and S9) [27]. For each Avr gene, 22–28% of the candidates could be annotated. The biological annotations of candidate avirulence genes are further discussed in the following section.

## 4. Discussion

The leaf rust fungus *Pt* is the most widely distributed wheat rust pathogen worldwide and is responsible for most of the wheat crop yield loss arising from rust [1]. Despite the significance of leaf rust, our understanding of *Pt*-wheat interactions related to pathogenicity as well as the genomic resources available for this pathogen remains limited. In the present study, we generated whole-genome sequence for 12 *Pt* isolates and RNA sequence for a further three *Pt* isolates and carried out an integrated study that simultaneously analyzed gene expression, SNP, InDel, and CNVs in relation to virulence profile, and identified 40, 64, and 69 candidates for *AvrLr1*, *AvrLr15*, and *AvrLr24*, respectively. This study has not only provided important new genomic resources for research into *Pt* but also established a practical framework of integrated analysis from multiple genomic aspects to explore candidate avirulence genes.

The high-quality genome assembly and annotation of the founding isolate Pt104 provides a good foundation for accurate genotyping [27]. Furthermore, careful selection of isolates with similar genetic background with contrasting virulence that differ only on one or two host R genes minimized differences not related to pathogenicity and greatly assisted the effective identification of candidate avirulence genes. *AvrSr50* was successfully identified using this approach, by comparing an isolate with virulence for *Sr50* (Pgt632) with a very closely related avirulent isolate Pgt279 [25]. This approach was similarly used in *AvrLr20* mining using 10 pairs of isolates differing in avirulence/virulence only to *Lr20* [23], and in identifying candidate avirulence genes in *P. hordei* for the resistance genes *Rph3*, *Rph13,* and *Rph19* using five stepwise mutant isolates within a single putative clonal lineage [28]. In this study, for *Lr1*, all four isolates S594, S625, S629, and S631 within the top clade of the phenotype dendrogram (Figure 1A) were selected. For *Lr24* and *Lr15*, pairwise comparisons were both constructed by virulent isolates (S547 and S523, respectively) and closely related avirulent isolates within the middle clade of the dendrogram. However, as the selected pairs for *Lr15* also contained covariates of *AvrLr20* and *AvrLr23* (Figure 1A), an additional pair of S365 vs. S563 from the bottom clade was added to diminish this cofounding effect.

Genome-wide comparisons were made for the 12 *Pt* isolates by mapping the Illumina sequencing reads of these pathotypes to the Pt104 genome. Across these isolates, the median mapping rate was 91% and the mapping rates ranged from 83% to 93% except for the isolate S523 (75%) (Table 1). The improvement of this mapping rate over that of our study on *AvrLr20* [23] based on the *Pt* Race 1 [58] reference genome is likely due to the higher quality of our Pt104 assembly and the closer relatedness of the isolates studied to the isolate used for the Pt104 reference.

Based on the genome-wide SNPs identified, a phylogenetic tree was constructed (Figure 1B). The overall topology of this phylogenetic tree is consistent with the dendrogram showing the relatedness of these isolates based on virulence (Figure 1A), with three clades comprising the same set of isolates. This observation fully supports the hypothesis that these isolates have evolved most likely by simple step mutations from founding isolates [15]. Furthermore, the three clades in the phylogenetic tree also showed consistent distinctions in mapping and variant statistics. For example, for each of the three clades from top to bottom (Figure 1B), the mapping percentages of the reference genome were about 98%, 99%, and 97%, respectively (Table 1), and the variant heterozygosity rates were about 75%, 92%, and 72%, respectively (Table 2). This consistency between the phylogeny and variants statistics clearly indicated that the inferred phylogenetic tree captured the overall genetic features across the 12 *Pt* genomes.

**Figure 5:**
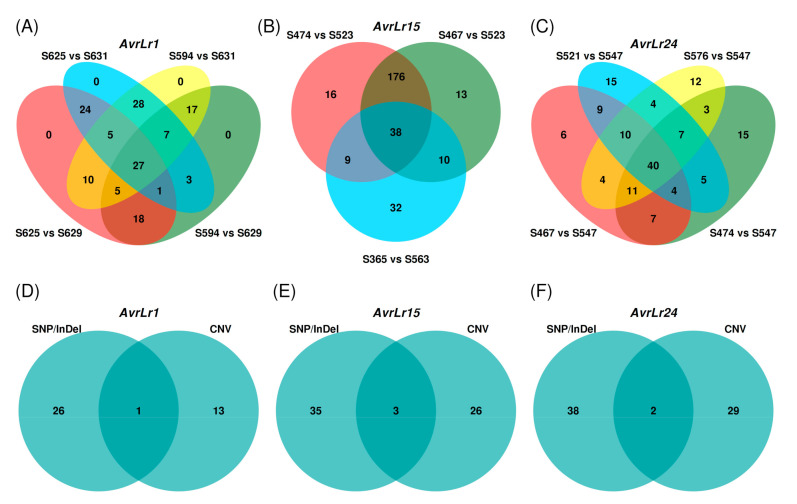
Venn diagrams for candidate avirulence genes identified by SNP/InDel and CNV. (**A**–**C**) demonstrate the intersection of candidates derived from SNP/InDel multiple pairwise comparisons for *AvrLr1* (27), *AvrLr15* (38), and *AvrLr24* (40), respectively. (**D**–**F**) demonstrate the final candidates combined from SNP/InDel comparison and CNV detection for *AvrLr1* (40), *AvrLr15* (64), and *AvrLr24* (69), respectively.

In the present study, the numbers of SNP and InDel variants detected across the *Pt* isolates were 451,414–606,306 and 71,807–80,629, respectively (Table 2). The total numbers of SNPs identified in this study are higher yet still comparable to the previously reported total numbers of SNPs ranging from 329,300 to 446,048 in a different panel of 20 *Pt* isolates [23]. Consistent with the improved mapping rate, the higher numbers of SNP detected are most likely due to the better quality of the Pt104 assembly and the closer relatedness of the isolates studied to the reference isolate. Out of the total variants identified, 72.4–93.2% were in heterozygous state (Table 2), which is consistent with our previous report for a panel of 20 *Pt* isolates with heterozygosity rate of 72–87% [23]. As aforementioned, among the 12 *Pt* isolates used here, the percentages of heterozygosity were similar within each clade of the phylogenetic tree and varied between clades. Thus, the higher end of 93.2% heterozygosity as compared to our previous study may reflect some intrinsic differences in the *Pt* genomes between various clades. By including both homozygous and heterozygous polymorphisms, the functional impact of the genomic variants was annotated (Table 3) and the subsequent analysis then focused on the SP genes harboring differential variants with functional impact (Supplementary File S4). Differential variants derived from pairwise comparisons based on contrasting virulence profiles led to the identification of 27, 38, and 40 candidates for *AvrLr1*, *AvrLr15*, and *AvrLr24*, respectively (Figure 5).

For Avr gene identification, there is no one-size-fits-all protocol as the exact mechanism underlying virulence acquisition is unknown and an Avr gene could be altered by any one or more of several diverse mechanisms such as amino acid mutation caused by SNPs and DNA segment deletion arising from CNVs. In pathogens of animals and plants, it has been shown that CNVs can be important contributors to pathogenic and phenotypic diversity such as virulence [29,59,60]. For example, CNVs at the *Avr1a* and *Avr3a* loci were detected among different strains of the oomycete *Phytophthora sojae* that were related to altered virulence and evasion of host immunity [61]. Given the significance of CNV in fungal pathogenicity, the present study integrated CNV analysis to complement our small genomic variants (SNPs and InDels) analysis for candidate avirulence gene mining.

The total CNVs detected across the *Pt* isolates ranged from 307 to 2235, and the median size of CNV was about 1800–2100 bp. Like SNP/InDel variation, the CNV statistics (Table 4) were distinct between the three clades in the phylogenetic tree. For example, for each clade from top to bottom (Figure 1A), the total CNV counts were about 2200, 300, and 1700, respectively and the percentages of bases of the reference genome were 7.5%, 1.7%, and 6.6%, respectively (Table 4). When SNP/InDel and CNV variations were inspected together (Table 2 and Table 4), the extend of CNV variation seemed to be correlated to the degree of SNP/InDel variation, both of which consistently showed distinct patterns between the clades of the phylogeny (Figure 1B). In contrast, a previous study on the genomic variation of wine yeast strains showed that although genetic diversity in the form of SNPs was low, CNV diversity was substantial and impacted on important biological functions associated with adaptation to the fermentation environment [30]. While this yeast study demonstrated that even when the SNP level is low, the CNV could be abundant, our study revealed substantial variation at both levels. Taken together, our and previous research have highlighted CNV as a substantial contributor to the genomic diversity, which warrants detailed examination independent of SNPs/InDels.

Out of the total CNVs identified, 44.8–50.2% CNVs spanned gene-encoding regions and 2.6–4.9% CNVs covered SP-encoding genes (Table 4). In total, 385–2039 genes were overlapped with CNVs per isolate. Although the total numbers and sizes of CNVs differed across the isolates, the percentage of CNVs overlapping either genes or SP genes was similar across the isolates (Table 4). For the identification of candidates for each Avr gene, within a comparison group CNVs were detected as differential when the copy number differed between avirulent and virulent isolates. For *AvrLr1*, *AvrLr15*, and *AvrLr24*, 14, 29, and 31 SP genes were identified as candidates, respectively (Supplementary File S6). Out of these CNV-derived candidates, only a couple overlapped with the SNP/InDel-derived candidates, which again implied that CNV could independently contribute to the genomic variations and thus should be integrated as an indispensable component for Avr gene mining. When the results from these two approaches were combined, the final number of candidates for *AvrLr1*, *AvrLr15*, and *AvrLr24* expanded to 40, 64, and 69, respectively (Table 5).

Effectors can be localized in the apoplast, cytoplasm, and nucleus. The locations of candidate effectors can be predicted using LOCALIZER, which can predict the locations of chloroplasts, mitochondria, and nuclei, and ApoplastP, which can differentiate between apoplast and non-apoplast locations. For the 40, 64, and 69 candidate effectors for *AvrLr1*, *AvrLr15*, and *AvrLr24*, the locations of 17, 33, and 34 were predicted, respectively. It was also noted that for each Avr gene only one or two candidate effectors had predicted locations by both LOCALIZER and ApoplastP, conflicting with each other. These conflicting predictions were tagged as “Uncertain” (Supplementary File S7).

Based on the InterPro annotation, we further inspected the biological functions of the candidate avirulence genes Supplementary File S9). For example, the candidate GN104ID162_017346 for *AvrLr24* was annotated with a Znf_TFIIS domain. TFIIS is a kind of eukaryotic transcription elongation factor that helps in synthesizing long RNAs [62]. GN104ID162_017346 was predicted as a nuclear effector by LOCALIZER, which was consistent with this functional annotation. GN104ID162_017346, identified by SNP/InDel, harbored three mutations: one SNP at position 920,126 and two frameshift insertions at positions 920,297 and 920,299 (Supplementary File S4). The two insertions only existed in the virulent isolate S547 in the four pairs (S467 vs. S547, S474 vs. S547, S521 vs. S547, and S576 vs. S547), which may account for the virulence of isolate S547. For the candidate avirulence genes identified in this study, all the annotation information generated (location, functional annotation, and effector probability) will facilitate biological validation studies in the future (Supplementary Files S8 and S9).

A limitation of this study that should be noted is that the isolate Pt104 on which the reference genome is based is virulent on *Lr1*, and the prediction of candidates for *AvrLr1* assumes that *AvrLr1* is only partially deleted in Pt104. In future studies, an isolate containing *AvrLr1* will be sequenced using long-read sequencing technologies to build a new reference genome assembly to validate the candidates for *AvrLr1*.

Given the complexity of fungal genomes and the limited knowledge of the genetic mechanisms underlying the development of virulence in fungal pathogens, more and more integrated approaches have been developed for effector detection. One such integrated approach is to combine genome-wide association analysis with variant comparisons, such as our study on *AvrLr20* mining [23], a previous study on *AvrPm3* in powdery mildew [63], and a recent study using 30 *Pst* isolates derived from ethyl methanesulfonate mutagenesis [21]. Although these association analyses still focus on genomic variations at the level of SNPs and InDels, our study is the first to integrate the major type of structure variation, CNV, into comparative analysis at the genome-wide scale in a rust pathogen. This CNV approach yielded candidates showing low levels of overlap with those derived from SNP/InDel, which again demonstrated that CNV was a relatively independent layer of genomic variation that could contribute to rust pathogenicity as discussed previously. In summary, integrative approaches that combine multiple layers of data analysis may more accurately explore candidate avirulence genes in the obligate biotrophic fungus.

## Figures and Tables

**Table 1:** Mapping information for the 12 *Puccinia triticina (Pt)* isolates.

Isolate	Total Reads (Quality Trimmed)	Reads Mapped to Reference	Percentage Mapped Reads	Average Coverage Fold	Percentage of Mapped Bases in Reference
S365	81,605,446	75,995,860	93.1%	72.7	98.2%
S563	72,359,456	66,985,626	92.6%	62.5	98.1%
S467	71,491,329	66,285,859	92.7%	62.0	99.4%
S474	66,268,039	57,035,893	86.1%	53.6	99.3%
S521	75,331,554	62,273,511	82.7%	58.2	99.3%
S523	75,802,433	56,814,472	75.0%	54.5	99.3%
S547	72,921,641	63,758,642	87.4%	59.9	99.3%
S576	69,188,369	63,626,559	92.0%	59.1	99.3%
S594	89,362,930	80,982,321	90.6%	75.5	97.8%
S625	69,718,296	64,161,304	92.0%	60.5	97.7%
S629	96,147,346	87,921,703	91.4%	81.7	97.9%
S631	78,547,125	69,753,222	88.8%	65.5	97.9%

**Table 2:** Summary of small genomic variants in the 12 *Pt* isolates.

Isolate	Total Variants	SNP	InDel	Insertion	Deletion	Heterozygous SNP	Heterozygous InDel	Percentage of Heterozygosity
S365	686,935	606,306	80,629	45,961	34,668	478,487	39,772	75.4%
S563	683,433	603,530	79,903	45,531	34,372	478,208	39,448	75.7%
S467	529,092	454,513	74,579	44,710	29,869	450,156	38,851	92.4%
S474	525,308	451,414	73,894	44,211	29,683	446,808	38,494	92.4%
S521	529,110	454,715	74,395	44,474	29,921	450,188	38,884	92.4%
S523	581,330	505,061	76,269	45,163	31,106	500,500	41,167	93.2%
S547	529,291	454,915	74,376	44,490	29,886	450,085	38,905	92.4%
S576	527,896	453,762	74,134	44,379	29,755	449,108	38,612	92.4%
S594	558,307	485,758	72,549	42,137	30,412	373,086	31,204	72.4%
S625	555,027	483,220	71,807	41,651	30,156	372,931	31,133	72.8%
S629	560,570	487,576	72,994	42,382	30,612	374,965	31,388	72.5%
S631	555,702	483,678	72,024	41,857	30,167	372,691	30,791	72.6%

**Table 3:** Summary of the functional impacts of small genomic variants in the 12 *Pt* isolates.

Isolate	Coding Variants	Percentage of Coding Variants	Genes Covered	Synonymous Variants	Nonsynonymous Variants	Frameshift Variants	Nonsense Variants
S365	104,269	15.2%	17,989	33,534	60,875	8136	1724
S563	103,663	15.2%	17,938	33,392	60,528	8036	1707
S467	80,511	15.2%	15,681	25,843	45,740	7734	1194
S474	79,896	15.2%	15,597	25,748	45,298	7634	1216
S521	80,514	15.2%	15,682	25,939	45,630	7747	1198
S523	88,416	15.2%	16,834	28,584	50,655	7788	1389
S547	80,496	15.2%	15,686	25,865	45,737	7688	1206
S576	80,238	15.2%	15,655	25,745	45,635	7647	1211
S594	84,957	15.2%	15,567	27,235	48,775	7521	1426
S625	85,022	15.3%	15,575	27,359	48,694	7543	1426
S629	85,340	15.2%	15,628	27,281	49,085	7544	1430
S631	84,818	15.3%	15,560	27,149	48,727	7516	1426

**Table 4:** Summary of copy number variations (CNVs) in the 12 *Pt* isolates.

Isolate	CNV Count	CNV Median Size (bp)	CNV Total Size (bp)	Percentage of Bases of Reference	Overlapping-Gene CNVs	Overlapping-SP Gene CNVs	Affected Genes	Affected SP Genes
S365	2231	1800	10,609,561	7.5%	1021	59	2039	69
S563	2235	1800	10,507,349	7.5%	1018	58	2014	67
S467	307	2100	2,381,715	1.7%	154	13	428	18
S474	324	2100	2,342,069	1.7%	155	12	432	17
S521	318	2100	2,202,295	1.6%	152	12	389	16
S523	324	1800	1,997,415	1.4%	149	10	385	16
S547	328	2100	2,418,223	1.7%	147	16	410	24
S576	310	2100	2,247,015	1.6%	152	13	407	18
S594	1713	2100	9,297,278	6.6%	819	52	1731	63
S625	1692	2100	9,263,378	6.6%	824	56	1736	68
S629	1688	2100	9,207,278	6.6%	804	54	1710	66
S631	1704	2100	9,066,903	6.5%	807	50	1692	61

**Table 5:** Summary of candidates identified by single nucleotide polymorphism (SNP)/insertion/deletion (InDel) and CNV for *AvrLr1*, *AvrLr15,* and *AvrLr24.*

Avr Gene	SNP/InDel Candidates	CNV Candidates	Overlapped Candidates	Final Candidates
*AvrLr1*	27	14	1	40
*AvrLr15*	38	29	3	64
*AvrLr24*	40	31	2	69

## Supplementary Materials

The following are available online at https://www.mdpi.com/2073-4425/11/9/1107/s1, Supplementary File S1: The virulence/avirulence profiles of 12 isolates on the catalogued resistance genes; Supplementary File S2: Mapping information for the three RNA samples; Supplementary File S3: Predicted SP genes; Supplementary File S4: Single nucleotide polymorphism (SNP)/insertion/deletion (InDel) comparison within SP genes for *AvrLr1*, *AvrLr15,* and *AvrLr24*; Supplementary File S5: SNP/InDel comparison within candidate avirulence genes for *AvrLr1*, *AvrLr15,* and *AvrLr24*; Supplementary File S6: Copy number variation (CNV) comparison. Sheet 1: CNV comparison summary. Sheets 2–4, 5–7, and 8–10 are for *AvrLr1*, *AvrLr15,* and *AvrLr24*, respectively. Sheet 2/5/8: List of CNVs. Sheet 3/6/9: List of genes overlapped by CNVs. Sheet 4/7/10: List of SP genes overlapped by CNVs; Supplementary File S7: Summary of locations of candidate avirulence genes; Supplementary File S8: Annotations for candidate avirulence genes for *AvrLr1*, *AvrLr15,* and *AvrLr24*; Supplementary File S9: Annotations with InterPro details for candidate avirulence genes for *AvrLr1*, *AvrLr15*, and *AvrLr24*.
